# Combined High-Dose LATTICE Radiation Therapy and Immune Checkpoint Blockade for Advanced Bulky Tumors: The Concept and a Case Report

**DOI:** 10.3389/fonc.2020.548132

**Published:** 2021-02-12

**Authors:** Liuqing Jiang, Xiaobo Li, Jianping Zhang, Wenyao Li, Fangfen Dong, Cheng Chen, Qingliang Lin, Chonglin Zhang, Fen Zheng, Weisi Yan, Yi Zheng, Xiaodong Wu, Benhua Xu

**Affiliations:** ^1^Department of Radiation Oncology, Fujian Medical University Union Hospital, Fuzhou, China; ^2^Department of Medical Imaging Technology, College of Medical Technology and Engineering, Fujian Medical University, Fuzhou, China; ^3^Department of Medical Imaging, School of Clinical Medicine, Fujian Medical University, Fuzhou, China; ^4^Department of Radiation Oncology, Thomas Jefferson Medical College, Philadelphia, PA, United States; ^5^Department of Medical Physics, Executive Medical Physics Associates, Miami, FL, United States

**Keywords:** lattice radiotherapy, high-dose LATTICE radiation therapy, immunotherapy, non-small cell lung cancer, bulky tumor, spatially fractionated radiotherapy

## Abstract

Although the combination of immune checkpoint blockades with high dose of radiation has indicated the potential of co-stimulatory effects, consistent clinical outcome has been yet to be demonstrated. Bulky tumors present challenges for radiation treatment to achieve high rate of tumor control due to large tumor sizes and normal tissue toxicities. As an alternative, spatially fractionated radiotherapy (SFRT) technique has been applied, in the forms of GRID or LATTICE radiation therapy (LRT), to safely treat bulky tumors. When used alone in a single or a few fractions, GRID or LRT can be best classified as palliative or tumor de-bulking treatments. Since only a small fraction of the tumor volume receive high dose in a SFRT treatment, even with the anticipated bystander effects, total tumor eradications are rare. Backed by the evidence of immune activation of high dose radiation, it is logical to postulate that the combination of High-Dose LATTICE radiation therapy (HDLRT) with immune checkpoint blockade would be effective and could subsequently lead to improved local tumor control without added toxicities, through augmenting the effects of radiation in-situ vaccine and T-cell priming. We herein present a case of non-small cell lung cancer (NSCLC) with multiple metastases. The patient received various types of palliative radiation treatments with combined chemotherapies and immunotherapies to multiple lesions. One of the metastatic lesions measuring 63.2 cc was treated with HDLRT combined with anti-PD1 immunotherapy. The metastatic mass regressed 77.84% over one month after the treatment, and had a complete local response (CR) five months after the treatment. No treatment-related side effects were observed during the follow-up exams. None of the other lesions receiving palliative treatments achieved CR. The dramatic differential outcome of this case lends support to the aforementioned postulate and prompts for further systemic clinical studies.

## Introduction

Lung cancer is the most commonly diagnosed cancer (11.6% of the total cases) and the leading cause of cancer death (18.4% of the total cancer deaths) based on the latest global cancer statistics (1). Non-small cell lung cancer (NSCLC) accounts for about 85% of all lung cancers, with approximately 40% of newly diagnosed NSCLC patients presented with stage IV disease (2, 3). Radiotherapy plays an important role in the definitive, preoperative and postoperative management of early stage and in the palliative treatment of advanced staged NSCLC. The use of radiation as a local ablative therapy is now recommended in the European Society for Medical Oncology (ESMO) guidelines for patients with stage IV disease who subsequently develop oligometastatic progression (4).

High ablative doses of local radiation therapy (RT), often referred to as stereotactic radiosurgery (SRS) or stereotactic body radiotherapy (SBRT) is usually applied to tumors with limited size (5). The application of SRS or SBRT in bulky tumors is often considered challenging due to the difficulties in controlling toxicities of the surrounding normal/critical organs. Spatially fractionated radiotherapy (SFRT) with GRID, proposed in 1909 and further developed since early 1950s, has been safely utilized for the treatment of bulky and advanced tumors with impressively accumulated clinical data (6–8). In recent years, the 2D GRID technique was extended to a 3D configuration, known as LATTICE radiotherapy (LRT) (9, 10). The safety and clinical efficacy of LRT has been reported in various voluminous tumors (11–15).

Based on the results of radiobiological studies, the possible mechanisms behind the effectiveness of SFRT have been attributed to certain bystander effects and abscopal effects, such as the radiation-mediated anti-tumor immunity (16–23) or perfusion modulation (10, 24). The key characteristics of SFRT in either GRID or LRT configurations is the Peak-Valley dose distribution, where high dose of radiation is delivered to the peaks or vertices, leaving relatively lower dose in the valleys (between the peaks or vertices) (9–15). In the context of immune modulation, as long as the peak dose is sufficiently high, although only partial volume of the tumor receives that high dose, the induced anti-tumor immunity can be expected and would subsequently contribute to the enhanced tumor control (25–30). As radiation-mediated immune activation follows the pathway of T-cell priming through antigen presentation, for immunogenic tumors, combining immune checkpoint blockades with high dose of radiation is a logical strategy and has been extensively studied with encouraging results (30–37). The recent studies also showed that combined ablative dose with low dose of radiation could lead to the reprograming of the immunosuppressive tumor microenvironment (TME) to become more immunogenic and synergistically augment the anti-tumor response (38). This is an important insight as SFRT intrinsically combines high and low dose in its Peak-Valley dose distribution. All these have suggested that HDLRT, when combined with checkpoint blockade immunotherapy could result in improved tumor control.

In the following case report, a patient with multiple metastatic lesions from a primary NSCLC received various regimens of palliative treatments, including conformal radiotherapy (CRT), intensity-modulated radiotherapy (IMRT), SBRT, LRT, chemotherapy, and immunotherapy. Remarkably, only one lesion treated with high-dose LRT (HDLRT) and anti-PD1 therapy achieved complete local response (CR).

## Case Presentation

A 33-year-old female patient initially presented with a lung mass in the right lower lobe, accompanied by cough for one month. She underwent video-assisted thoracoscopic right lower lobectomy and systemic mediastinal lymph node dissection as curative intent resection on May 10, 2017. The surgical histopathological report demonstrated diagnosis of invasive adenocarcinoma in the lower lobe of the right lung. The lung mass measured 7, 4.5, and 3.2 cm in the greatest dimensions. Post-surgical staging was T3N2M0. Postoperatively, she received two cycles of adjuvant chemotherapy of PP regimen (pemetrexed disodium 0.8 g dl + cisplatin 0.4g dl) from June to August 2017.

In September 2017, the patient developed metastatic disease in L2-3 spine diagnosed by magnetic resonance imaging scan. Computed Tomography (CT) scan showed multiple metastases of different sizes in both lungs, a metastatic nodule in thyroid, and a mass in the posterior chest wall measuring 2.0 cc with maximum dimensions 1.8x1.7x1.2 cm. Single-Photon Emission Computed Tomography (SPECT) scan also showed multiple metastases in right parietal bone, cervical vertebra, L2-3 spine, left ilium, and right sacroiliac joint.

**Figure 1:**
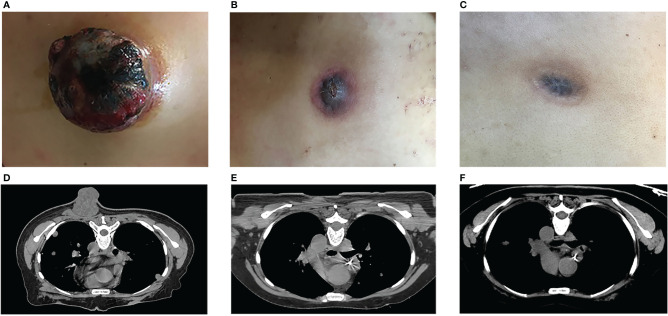
Tumor response to treatment. The metastatic mass in the posterior chest wall, before **(A, D)** and after **(B, C, E, F)** HDLRT on October 18th. (Permission by Radiation Research to extract from Figure 3 in Wu X et al. (10). © 2021 Radiation Research Society).

The patient was found to have EGFR exon 20 insertion mutation, ALK and ROS-1 negative and over 70% expression of PD-L1. The patient started the first cycle of checkpoint inhibitor therapy using Pembrolizumab, an anti-PD-1 monoclonal antibody (100mg ivgtt d1 q3w) on September 30th, 2017. While the treatment was on going, the metastatic mass in the posterior chest wall grew rapidly from 2.0 cc to 63.2 cc with maximum dimensions 5.0 × 5.4 × 5.3 cm on October 10, 2017, in less than a month, (**Figures 1A, D**). The lesion was ulcerated with slight local bleeding. New metastases in brain and right sternoclavicular joint were subsequently observed on MRI and CT images.

### High-Dose LATTICE Radiation Therapy (HDLRT)

The decision was to treat the fast-growing posterior chest wall mass with HDLRT to combine with the on-going anti-PD-1 treatment.

Treatment planning was performed on the MULTIPLAN (Accuray, Incorporated, Sunnyvale, CA). A CyberKnife VSI Robotic Radiosurgery System (Accuray, Incorporated, Sunnyvale, CA) was used for delivering a single fraction of LRT with 20 Gy prescribed to six high-dose vertices.

**Figure 2:**
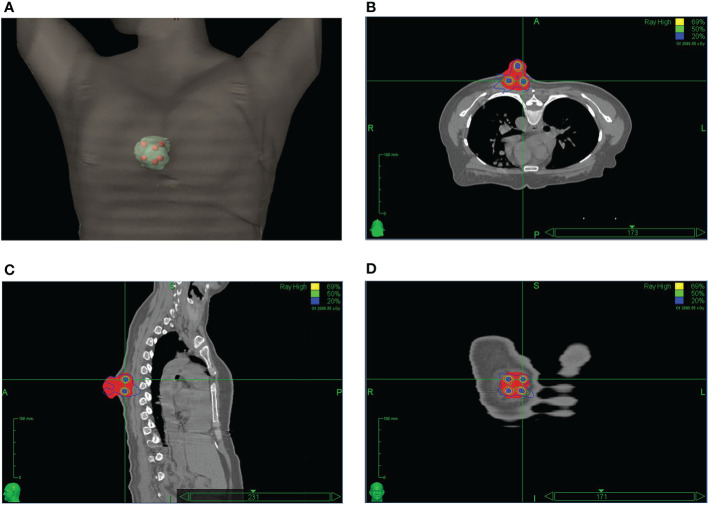
The LATTICE dose distribution in a 3D view **(A)**; in an axial plane **(B)**; in a sagittal plane **(C)**; and in a coronal plane **(D)**. The high-dose vertices (total of six) received 20 Gy to the 69% isodose line. The doses between the dose-vertices (valley) were in the order of 25% of the maximum (peak) dose. (Permission by Radiation Research to extract from Figure 3 in Wu X et al. (10). © 2021 Radiation Research Society).

**Figure 3:**
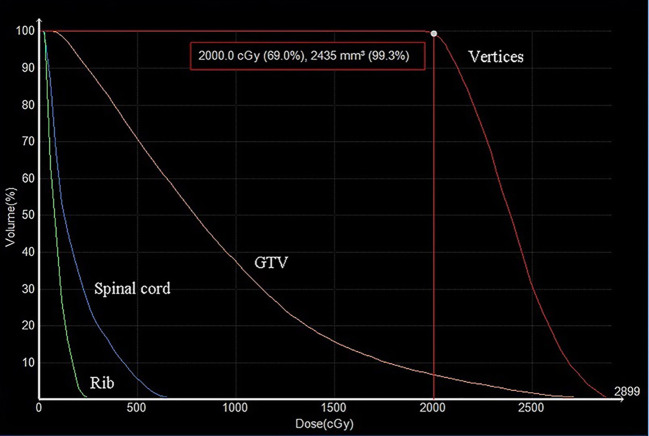
The dose-volume histograms (DVHs) of high dose-vertices, GTV, and normal tissue. D99.3% of the vertices was 20 Gy. The doses to the ribs and spinal cord were effectively minimized.

**Figure 4:**
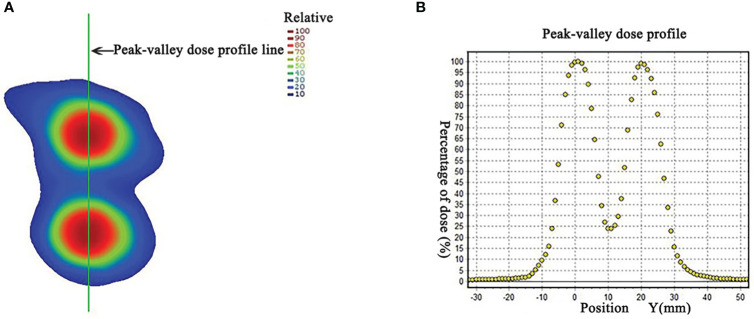
**(A)** Dose distribution in a coronal plane. The peak-valley dose profile **(B)** along the green line marked in panel **(A)**, showing the peak to valley dose ratio of about 4. (Permission by Radiation Research to extract from Figure 3 in Wu X et al. (10). © 2021 Radiation Research Society).

The HDLRT was configured with six spherical high dose vertices with diameter of 1.0 cm distributed within the GTV and with 2.0 cm of separation (center to center). The optimized plan resulted in the doses covering 98%, 95%, 50%, and 5% of the vertices volume (D98, D95, D50, and D5%) being 20.95, 21.40, 24.88, and 27.85 Gy, respectively; the maximal dose of the spinal cord and rib being 2.67 and 7.45 Gy, respectively. The dose distribution is shown in **Figure 2**. The dose-volume histograms (DVHs) of the high-dose vertices, GTV, ribs, and spinal cord were shown in **Figure 3**. The Peak-to-Valley dose profile (**Figure 4**) showed the valley dose between vertices to be about 25% of the peak dose. Note that only 6.5% of the GTV received the prescribed vertex dose of 20 Gy and higher, and that the DVH of the GTV is closely similar to that of the published data with GRID.

The HDLRT was given on October 18, 2017, 18 days after the initiation of the first cycle of Pembrolizumab.

### Other Treatments

Subsequent to the HDLRT, from October 30, 2017, to March 28, 2018, patient continued to receive another six cycles of Pembrolizumab (100 mg ivgtt d1 q3w, for each cycle).

Additionally, the patient received SBRT with 10 Gy in two fractions, 20 Gy in 1 fraction, and 15 Gy in 1 fraction for a metastatic tumor at the L2-3 spine, a right lung metastasis near anterior chest wall, and a tumor at the T10 spine, respectively; CRT with 8 Gy in two fractions and 20 Gy in five fractions for a spinal metastatic tumor at the C3-5 levels and the whole brain, respectively; IMRT with 30 Gy in 10 fractions for a thyroid and a posterior sternal metastasis, and 8 Gy in four fractions for the metastatic abdominal lymph nodes, respectively; LRT with 12 and 10 Gy in 1 fraction for the spinal metastatic tumors at the L3 spine and psoas, and the metastatic abdominal lymph nodes, respectively. From February 2018 to May 2018, the patient also received four cycles of VEGF targeted therapies with bevacizumab and three cycles of chemotherapy with gemcitabine. The timeline and therapeutic interventions were listed in **Supplementary Table 1**.

### Clinical Outcome

While all metastatic lesions responded to various palliative treatments, only the posterior chest wall metastatic tumor achieved complete response. Under the background of anti-PD1 treatment, the tumor regressed 77.84% over one month after the HDLRT and then continued to shrink. Two months after the HDLRT, in addition to further shrinkage, all symptoms were relieved with the bleeding/discharging totally under control. This posterior chest wall tumor achieved complete local response (based on visual and radiographic exams) five months after the HDLRT without side effects (**Figures 1B, C, E**, **F**).

CT scans of the chest and abdomen on May 10, 2018 showed progression of multiple metastases in both lungs, mediastinum, retroperitoneum, right lower pleura, left upper middle abdominal cavity, double ilium, uterus, and T10 spine. Metastases in the right thyroid, pancreatic neck, bilateral adrenal glands, both kidneys, L3 spine and psoas, and C3-C5 spine were stable. Metastases in the right sternoclavicular joint and right lung near anterior chest wall shrank moderately. Due to the subsequent progression of metastases in multiple sites with cancerous fever and abdominal infection, the patient finally succumbed to the disease, seven months after the HDLRT. The treatment site of the posterior chest wall remained disease-free until patient’s death.

## Discussion

Radiation therapy when used for palliative management of advanced cancers employs either conventional fractionation or SBRT regimens with dose lower than that of definitive, curative treatments, and would expectedly result in partial tumor response. LRT when used as palliative treatment would also lead to partial response in general. LRT as a safe boost to conventionally fractionated radiotherapy had shown clinical success in a variety of bulky tumors such as advanced gynaecological tumors and voluminous lung tumors (11–15). The first patient with locally advanced lung cancer treated with LRT followed by conventionally fractionated radiotherapy, combined with chemotherapy demonstrated excellent clinical response after 6 years follow-up (13). Since 2010, over 150 patients have been treated with LRT and more reports of clincal outcomes are anticipated (10). However, to this day, LRT alone as induction of anti-tumor T-cell immunity, to combine with immune checkpoint blockade treatment has not been reported.

In the reported case, with the anti-PD1 treatment in parallel, except for the posterior chest wall lesion that received HDLRT of 20 Gy, all other lesions achieved only partial response, including the ones treated with SBRT of 20 Gy in a single fraction (full tumor coverage), and LRTs with 10 Gy and 12 Gy of vertex doses. This implies that not only a high dose (20 Gy or higher) is essential; the spatial fractionation with Peak-Valley or High-Low dose alternation within the tumor volume might also be critical to mediate effective anti-tumor immune response. This is consistent with a number of research works favoring high dose for effective anti-tumor T-cell priming (20, 21, 25, 30), and that when combined with low-dose treatment, radiation-induced immune modulation might be augmented (38, 39). Additionally, it has been postulated that the low dose regions (valleys) might preserve the perfusion needed for circulating the factors essential for anti-tumor immunity (10, 40–44).

It is worth noting that, with only 6.5% of the GTV receiving the dose of 20 Gy and higher, the effective uniform dose (EUD) of the GTV was calculated to be 1.2 Gy, using Niemierko’s phenomenological model (45) with a=−10 (typically suggested for tumors). Based on the traditionally understood mechanism of radiobiology, the probability of achieving complete local control with such a dose for a tumor of 63 cc would be nearly zero. Given the fact that this tumor was not responsive to the initial anti-PD1 treatment and none of the other tumors showed significant reduction throughout the curse of the treatments, the synergetic effect of combining HDLRT with anti-PD1 becomes a plausible speculation. To summarize the postulated mechanism, in HDLRT the dose in the vertices are sufficiently high (>20 Gy) to induce neo-antigen release and initiate the cascade of APC (antigen presenting cell)-based T-cell priming; the dose in between the vertices is low enough to preserve internal tumor circulation/perfusion to potentially facilitate the infiltration of APCs and the primed cytotoxic T-cells; the highly heterogeneous dose configuration could reprogram the immunosuppressive TME to become more immunogenic; and when synergistically treated by checkpoint inhibitors, the primed T cells could attack tumor cells without being exhausted.

Mohiuddin et al. treated a pembrolizumab-refractory patient with locally advanced melanoma who was dramatically re-sensitised to the same drug by the administration of parallel opposed, spatially fractionated GRID radiation therapy. Their result suggested the similar synergistic effect of high-dose GRID radiation therapy as a primer for immunological response (46). Our finding echoes with their result. However, abscopal response of other tumors with the appreciable magnitude was not observed in this case study.

## Conclusion

SFRT, with its long history of evolution is currently gaining new momentum and much of the new potentials are awaited for further exploration (47). LRT can safely deliver potentially immunogenic high dose to partial volume of bulky tumors. When combined with immune checkpoint blockades, therapeutic effects greater than traditional palliation/de-bulking, and even complete local tumor eradication are possible. The reported case showed the dramatic difference in tumor response between HDLRT and an array of palliative radiation therapy regimens when combined with anti-PD1 immunotherapy in a same individual, suggesting such strategy of combining HDLRT and immune checkpoint blockades might present a universally applicable treatment option if the clinical efficacy and safety can be systemically tested and proven.

## Data Availability Statement

The original contributions presented in the study are included in the article/**Supplementary Material**. Further inquiries can be directed to the corresponding authors.

## Ethics Statement

The studies involving human participants were reviewed and approved by Ethical Committee of Fujian Medical University Union Hospital. The requirement for informed patient consent was waived by Ethical Committee of Fujian Medical University Union Hospital due to the retrospective nature of this study.

## Author Contributions

BX and XW are responsible for study design. BX, XW, LJ, and XL, contributed to the writing of the manuscript. LJ, XL, JZ, and WL are responsible for developing treatment plan, data analysis, and contributed to the physics portion of the manuscript. FD, CC, QL, CZ, and FZ are responsible for clinical data collection and contributed to the clinical portion of the manuscript. WY and YZ contributed to data analysis discussion and manuscript editing. All authors contributed to the article and approved the submitted version.

## Funding

This work was supported by the Youth Teacher Program of Education Department of Fujian Province, Fuzhou, Fujian, China [grant JAT 170223] and the Fujian Provincial Department of Finance, Fuzhou, Fujian, China [grant 2018B055].

## Conflict of Interest

YZ and XW were employed by Executive Medical Physics Associates.

The remaining authors declare that the research was conducted in the absence of any commercial or financial relationships that could be construed as a potential conflict of interest.
