# Supplementary material for: Combined High-Dose LATTICE Radiation Therapy and Immune Checkpoint Blockade for Advanced Bulky Tumors: The Concept and a Case Report
Source: Front Oncol. 2021 Feb 12;10:548132. doi: 10.3389/fonc.2020.548132 (PMC7907519; doi:10.3389/fonc.2020.548132)
Supplement: Supplementary file 1 [file Table_1.docx]

Supplementary Material

# Supplementary Table

**Supplementary Table 1** **Treatment timeline and relevant therapeutic interventions.**

| **Time** | **Therapeutic interventions** |
| --- | --- |
| May 10, 2017 | Video-assisted thoracoscopic right lower lobectomy and systemic mediastinal lymph node dissection |
| June 9, 2017 to August 15, 2017 | Adjuvant chemotherapy with 3 cycles of pemetrexed 800 mg and cisplatin 400 mg on Day 1 (21 days per cycle) |
| September 27, 2017 | SBRT with 10 Gy in 2 fractions for spinal metastatic tumor at the L2-3 spine |
| September 30, 2017 | Immunotherapy with pembrolizumab 100 mg on Day 1 of Cycle 1 (21 days per cycle) |
| October 18, 2017 | HDLRT with 20 Gy in 1 fraction for metastasis in posterior chest wall |
| October 23, 2017 to January 11, 2018 | Immunotherapy with pembrolizumab 100 mg on Day 1 of Cycle 2-5 (21 days per cycle) |
| October 27, 2017 | 3D-CRT with 8 Gy in 2 fractions for spinal metastatic tumor at the C3-5 spine |
| November 6, 2017 | 3D-CRT with 20 Gy in 5 fractions for whole brain |
| November 20, 2017 | SBRT with 20 Gy in 1 fraction for a right lung cancer metastasis near anterior chest wall |
| December 6, 2017 | LRT with 12 Gy in 1 fraction for spinal metastatic tumor at L3 spine and psoas |
| December 27, 2017 | IMRT with 30 Gy in 10 fractions for thyroid and posterior sternal metastasis |
| December 28, 2017 | SBRT with 15 Gy in 1 fraction for spinal metastatic tumor at L3 spine |
| January 24, 2018 | IMRT with 8 Gy in 4 fractions for metastatic abdominal lymph nodes. |
| February 8, 2018 to March 3, 2018 | Immunotherapy with pembrolizumab 100 mg on Day 1 of Cycle 6-7 (21 days per cycle) |
| February 6, 2018 to March 2, 2018 | Targeted therapy with 2 cycles of bevacizumab 400 mg and chemotherapy with docetaxel 120mg on Day 1 (21 days per cycle) |
| March 28, 2018 | Immunotherapy with pembrolizumab 100 mg on Day 1 of Cycle 8 (21 days per cycle) |
| March 24, 2018 to April 25, 2018 | Targeted therapy with 2 cycles of bevacizumab 400 mg on Day 1 and chemotherapy with gemcitabine 1600 mg on Day 1 and 8 (21 days per cycle) |
| May 8, 2018 | LRT with 10 Gy in 1 fraction for metastatic abdominal lymph nodes |

Abbreviation: SBRT: stereotactic body radiotherapy; HDLRT: high-dose LATTICE radiation therapy; LRT: LATTICE radiation therapy; 3D-CRT: 3D conformal radiation therapy; IMRT: Intensity-modulated radiation therapy
